# Novel Approach and Interpretation for the Determination of Electromagnetic Forming Limits

**DOI:** 10.3390/ma13184175

**Published:** 2020-09-19

**Authors:** Koray Demir, Siddhant Goyal, Marlon Hahn, Erman Tekkaya

**Affiliations:** 1 AutoForm Engineering Deutschland GmbH, Marktstraße 46, 88212 Ravensburg, Germany; koray.demir@autoform.de; 2 Institute of Forming Technology and Lightweight Components (IUL), TU Dortmund, Baroper Strasse 303, 44227 Dortmund, Germany; marlon.hahn@iul.tu-dortmund.de (M.H.); Erman.Tekkaya@iul.tu-dortmund.de (E.T.)

**Keywords:** electromagnetic forming, forming limit diagram, formability, impulse forming

## Abstract

A new method to determine electromagnetic forming limits curves (EM-FLCs) for sheet metals is proposed. The different strain paths (between uniaxial and biaxial tension) are achieved by specific tool coil and specimen designs. It is ensured that the apex of the specimen deforms on a constant strain path, and excess bending at the apex is avoided. This is done so that the determined EM-FLCs are comparable to their quasi-static counterparts. The method determines the EM-FLCs for the aluminum alloys AA-1050a-H24 and EN AW-5083-H111 and the magnesium alloy Mg AZ31-O. Overall, it is observed that the necking limits in electromagnetic forming (EMF) are higher compared to quasi-static forming. The fracture surfaces of electromagnetically deformed specimens are examined to reveal the existence of out-of-plane shear stresses. A numerical analysis corroborates this observation and their variation with strain rate. The presence of such stresses is proposed as a possible reason for the increased necking limits in EMF. As reasons for higher forming limits, previous research has identified inertial stabilization, strain rate hardening, die impact, and change in deformation mechanism. The current study reaffirms the positive effect of inertial stabilization and makes key observations in the increase of twinning in EMF of Mg AZ31-O.

## 1. Introduction

The prime intention to determine the forming limits curves (FLCs) is to have a robust failure (necking) criterion for sheet metal forming for a particular strain rate. The standard Nakajima test is performed at quasi-static conditions for various strain paths. Several attempts for the determination of FLCs for high-speed processes are found in the literature, which observed the change in material properties of various alloys at higher strain rates. The high-speed FLCs can be used in the Finite Element Method (FEM) for process design and failure prediction. 

### 1.1. Forming Limits in High-Speed Processes

Forming limits higher than those in the quasi-static strain regimes have been observed in the literature and are not limited to electromagnetic forming. To determine the increased forming limits, some researchers conducted bulging experiments with full or tensile test specimens. Oliviera et al. [1] let full AA-5754 specimens bulge into a rectangular die opening. Even though the requirements of FLC determination were not fulfilled (constant strain path, no excessive bending), the apex strains were observed to be above the quasi-static FLC. 

**Figure 1:**
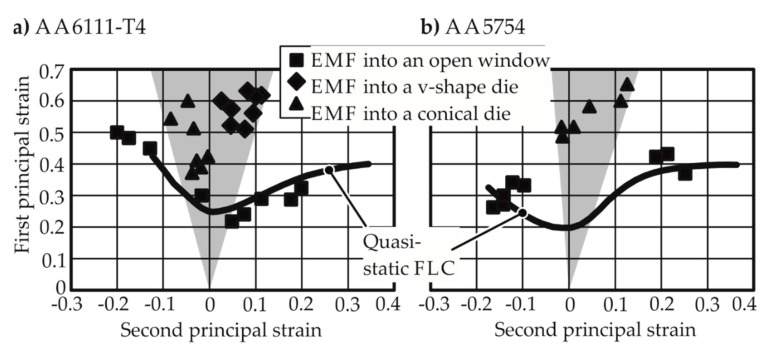
Forming limits reached by Golovashchenko [3] via electromagnetic forming. (**a**) Higher forming limits observed in the closed die forming cases for AA 6111-T4; (**b**) Marginally higher formability obtained for AA 5754 in open die forming.

Imbert [2] attempted to create a biaxial strain state by letting full AA-5754 and AA-6111 specimens bulge into a round die opening for free-forming experiments and also into a closed die. It was observed that higher strains in the apex were observed only for the EMF process where the sheet was formed into a die. In free EMF, the results could not exceed the quasi-static FLC. Golovaschenko [3] made a similar observation when strain results higher than the quasi-static FLC were observed after the introduction of female dies, as shown in Figure 1. The range of the results for higher strain rates obtained using the die is shown in a gray field. 

Spiral, straight, and uniform pressure actuators have been employed for EMF. A spiral coil is unable to apply pressure at the center and is unsuitable for specimen geometries having cutouts, as is the case for uniaxial and plane strain cases. A straight coil cannot apply a smooth and uniform pressure distribution. Furthermore, the induced current has no defined path to complete the circuit. A ‘uniform pressure actuator’ (UPA) proposed by Kamal [4] and tested by Jimbert et al. [5] can produce nearly uniform workpiece acceleration. A disadvantage of such a setup is the current transfer between multiple conductors during the process and possible arcing near the workpiece. The tooling for UPA is rather complex and leads to relatively long preparation times for a single experiment [5]. 

**Figure 2:**
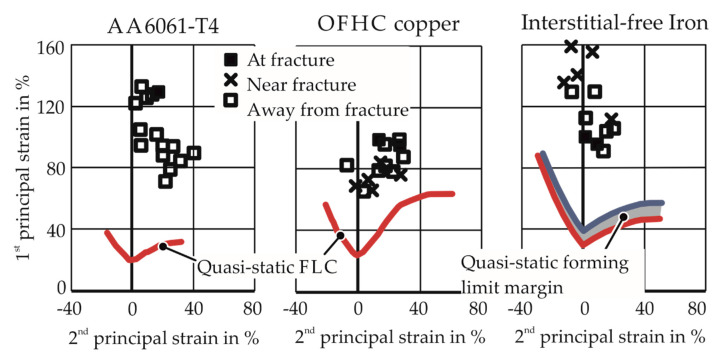
Forming limits observed by Balanethiram and Daehn [6,7]. Free electrohydraulic forming into a round die opening.

Similar observations were made for other impulse-forming processes as well. To examine higher forming limits in electrohydraulic forming, Balanethiram and Daehn [6,7] bulged full specimens into a round die opening, with estimated strain rates to be around 1000 s^−1^ for various materials; see Figure 2. The experiments resulted in strains much higher than the quasi-static forming limits. Maris [8] obtained apex fracture for four different strain paths for higher strain rates in electrohydraulic forming through careful numerical design of the pressure chamber and specimen shapes. The numerical modeling showed that the strain paths achieved were constant.

To examine higher forming limits in explosive forming, Wood [9] conducted dome bulging experiments using high explosives in a liquid medium. The 17–7 PH stainless steel specimens showed 73% increase in forming limits while other materials, such as molybdenum and vanadium–chromium–titanium alloy, did not show any increase. Furthermore, Wood [9] reported that the increase in formability is observed when a certain workpiece velocity is exceeded.

### 1.2. Problems in Determining Forming Limits in High-Speed Processes

In summary, the following problems are observed in the determination of forming limits at higher strain rates:

1. Difficulty in achieving constant strain paths: Due to the use of “conventional” tools such as straight or spiral coils, a uniform pressure distribution is not achieved in the specimen, leading to bending and non-constant strain paths.
2. Failure/fracture in regions apart from the apex of the specimen: It is expected for the FLC determination that the peak strains in the specimens occur at the apex. However, in many instances, failures occur at regions other than the apex, which lead to a non-constant strain path. This can be observed in the results of Li et al. [10], where failure is seen at the die entry radius. In electrohydraulic forming, Rohatgi et al. [11] observed fracture at the ligament in cruciform-shaped specimens. In explosive forming, Wood [9] explains that after the third critical velocity, the fracture observed in the specimens is primarily circumferential.
3. Strain rate during the determination of the FLC: In electrohydraulic forming, the results produced by Maris et al. [12] show higher FLCs. However, the points on the higher FLC come from different experiments with different strain rates. As the quasi-static FLC is produced at a constant strain rate, the points for an FLC in high-speed processes should also be determined for specific strain rates, e.g., at least identical average strain rates.

### 1.3. Possible Reasons for Higher Forming Limits in Impulse Forming

#### 1.3.1. Die–Sheet Interaction

**Figure 3:**
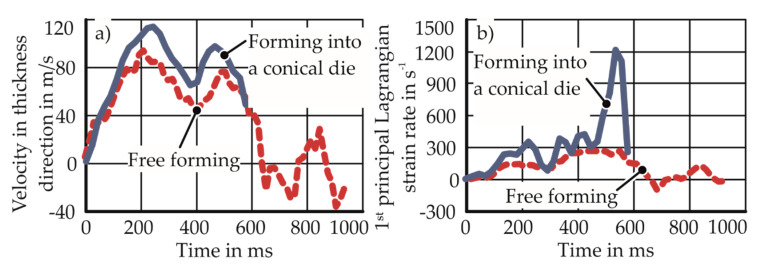
Electrohydraulic forming into a round die opening, with a conical die (in blue) and without a conical die (in red) (Rohatgi et al. [13]). Measurements are from the specimen apex. The conical die angle is 84°. (**a**) Increased specimen velocity due to the die; (**b**) increased strain rate due to the die.

In the research conducted by Imbert [2], Golovashchenko [3], Rohtatgi et al. [13], and Jenab et al. [14], enhanced formability was observed in the presence of a die during the process. Rohatgi et al. [13] explains that the changed process kinematic due to the die cavity leads to an increase in the velocity and strain rate. The variation of the principal strain rate with time for free forming and die forming is shown in Figure 3. 

Jenab et al. [15] claims that the reason for the higher forming limits is the through-thickness, out-of-plane compressive stress generated by the sheet–die impact. Imbert [2] explains the higher forming limits in forming with a die as the resultant of the emergence of intense bending–unbending behavior due to the impact. This causes high compressive stresses and decreases void growth.

#### 1.3.2. Inertial Stabilization

**Figure 4:**
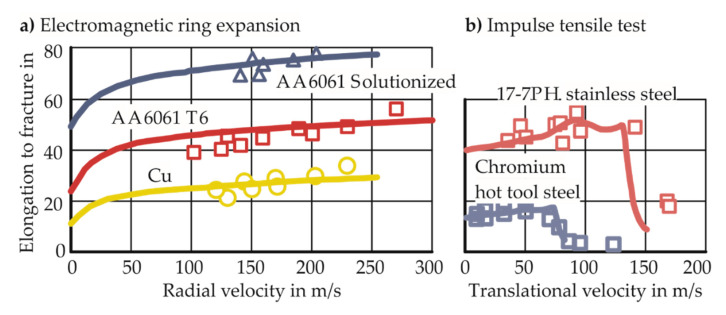
Lines show the simulation results of Hu and Daehn [16]. Points show the experimental results of (**a**) Altynova et al. [17] and (**b**) Wood [9].

Hu and Daehn [16] considered high-speed tensile tests in numerical simulations. The one-dimensional dynamic simulations use a temperature and strain-rate insensitive constitutive material model. The increase in formability observed experimentally by Altynova et al. [17] and Wood [9] could be successfully modeled in the simulations. As no other effects apart from the dynamic nature of the problem were considered in the simulations, the improvement in formability was attributed to inertia. The results of their analyses are shown in Figure 4. Regazzoni et al. [18] explain the effect of inertia as the continued deformation outside the neck, which is not observed in the quasi-static cases (after the onset of necking, the deformation outside the neck stops instantaneously) and increases the elongation to fracture. In this way, inertia delays the evolution of necking.

#### 1.3.3. Strain Rate Hardening

Thomas and Triantafyllidis [19] conducted a Marciniak–Kuczynski [20] analysis of the electromagnetic forming (EMF) process for AA-6061-T6. For the numerical analysis, the flow stress was assumed to be a power function of the effective plastic strain *ε*, the plastic strain rate $\dot{\varepsilon }$, and the temperature *θ*, as shown in Equation (1):

(1) $$\sigma \;\left(\varepsilon ,\dot{\varepsilon },\theta \right)=\;\sigma_{y}\;{\left(1+\frac{\varepsilon }{\varepsilon_{y}}\right)}^{n}{\dot{\varepsilon }}^{m}{\left(1+\left(\frac{\theta -\theta_{0}}{\theta_{m}-\theta_{0}}\right)\right)}^{\alpha }$$

where the strain rate sensitivity parameter *m* is dependent on temperature. In the analysis, higher formability was revealed in electromagnetic forming, which was attributed to the change in constitutive behavior (strain rate hardening), as inertial effects were ignored. Hadianfard et al. [21] conducted quasi-static and high-speed tensile tests for aluminum alloys AA-5182 and AA-5754. To explain the higher elongation to fracture in the high-speed tests, they showed the negative strain-rate sensitivity in the quasi-static range and positive strain rate sensitivity for AA-5754 at strain rates up to 1500 s^−1^. In the dynamic range, strain localization leads to a higher strain rate at which material hardening is observed, which slows down the localization and delays failure. 

#### 1.3.4. Change in Failure Mechanism

Hadianfard et al. [21] detected several large intense shear bands in quasi-static tensile tests of AA-5182 with a large number of voids, micro-cracks, and damaged second phase particles in the bands. Outside the bands, the damage was observed to be relatively less. However, the damage was primarily located outside the shear band for the high-speed specimens. Based on these observations, they concluded that quasi-static forming led to ductile shear fracture, while high-speed forming led to ductile tensile fracture. In compression tests, a change in the deformation mechanism from slip to twinning at high strain rates was observed previously by Ferreira et al. [22], Ulacia et al. [23], and Li et al. [24]. 

### 1.4. Purpose of the Work

To determine the possible reasons for higher forming limits in EMF, a novel method to determine the EM-FLCs experimentally is needed. The current work intends to develop the method to determine such EM-FLCs while following the same requirements of quasi-static FLCs. Such a method is presently non-existent in the literature. The objectives of the tests to be developed are:

- To deform the specimens on constant strain paths between uniaxial and biaxial tension under free-forming conditions
- To deform the specimens without excessive bending
- To achieve failure in the apex of the specimens and avoiding failure at the periphery, specimen cutouts, or die radius
- Ensure identical average strain rates for different points of a particular FLC.

Once the EM-FLCs are obtained, they are discussed and compared to their quasi-static counterparts. The study aims to explore the reasons for differences between the limits of quasi-static and electromagnetic forming limits. This analysis is undertaken through two approaches, namely numerical modeling and micrographic analysis.

## 2. Materials and Methods

### 2.1. Numerical Model for Experimental Design

The tool coil and specimen design are essential if the problems with FLC determination in high-speed processes observed in the literature are to be avoided. The numerical modeling was used to predict the strain concentration, applied magnetic pressure, etc. to develop the coils and specimen forms for the experiments. In the model, a measured current curve is provided as the input. The model is created in LS-Dyna, where the electromagnetic solver is loosely coupled with the thermal–mechanical solver. The specimen and the coil provided temperature-dependent thermal and electrical properties such as the thermal conductivity, heat capacity, and electrical conductivity, but the mechanical properties of the specimen are neither strain-rate nor temperature-dependent, as only quasi-static flow curve data were available from tensile tests. The coils are modeled as rigid bodies. The air between the various elements was modeled using the boundary element method (BEM) to avoid the high number of elements and remeshing when the air mesh faces high deformation. 

#### 2.1.1. Specimen Design

**Figure 5:**
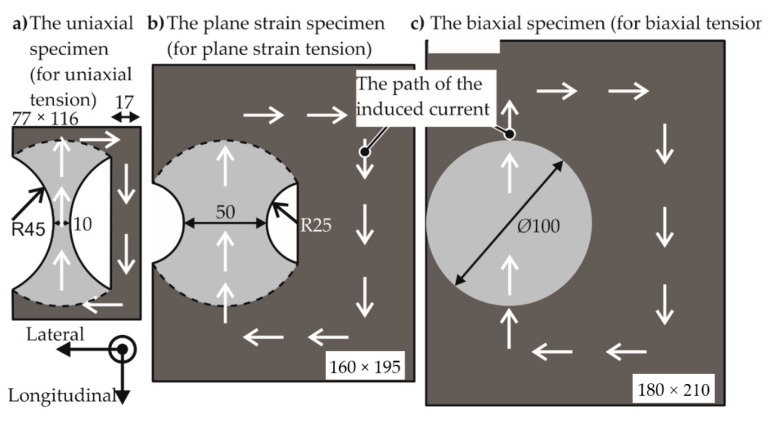
The three specimen shapes of the electromagnetic forming limit test. The brighter shade represents the deforming region. The darker shade acts as a return path for the induced current to close itself. Dimensions in mm.

Three different specimen shapes were designed to implement three different strain paths, namely uniaxial tension, plane strain tension, and biaxial tension. For the application of uniform pressure on the specimen, it is important for the specimen to have a uniform induced current with a path to complete the circuit. To this effect, the specimen geometry was provided with an external return path to complete the circuit for the induced current, as seen in Figure 5. This current path was also observed in the numerical model. 

**Figure 6:**
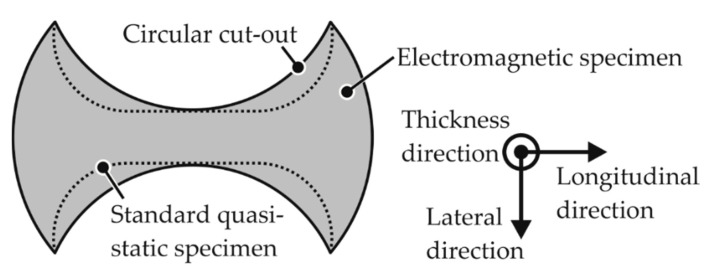
The electromagnetic forming limit test replaces the standard doge-bone shape with circular cutouts in the deforming region of the specimens.

The regions intended to be deformed are based on the standard specimens for the quasi-static forming limit test, which were designated by ISO/DIS 12004–2 [25]. Some modifications based on the observations made in the numerical model and applied on the standard specimens are:

1. The EM-FLC determination process prescribes circular cutouts instead of the dog-bone shape of the standard specimen to prevent a double neck, which was a phenomenon first observed by Wood [9] in high-speed forming. A sample specimen is shown schematically in Figure 6.
2. The standard specifies an Ø105 mm die opening and a Ø200 mm specimen diameter.
3. The electromagnetic forming limit test halves these values to reduce the required voltage to let the specimen fracture within the capacity of the electrical equipment.

#### 2.1.2. Coil Design 

**Figure 7:**
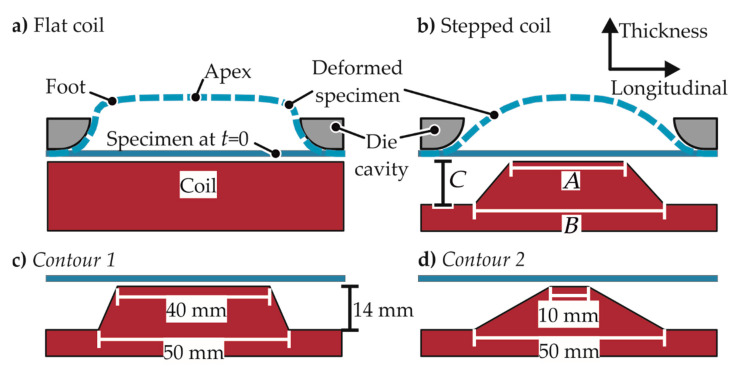
A coil that is flat in the longitudinal direction. (**a**) Typically used flat coil with bending at the foot; (**b**) A stepped coil; (**c**,**d**) two of the stepped contours used in the simulations.

Unlike a flat coil, which applies equal pressure on the foot of the specimen as it does on the apex, the coil is designed to focus the pressure on the apex. For this purpose, a stepped contour is used, which is optimized to acquire the least amount of bending in the specimen. The designs for the coil for the different strain paths are shown in Figure 7. 

This optimization is performed using the numerical analysis where the bending of the specimen around the longitudinal axis is examined. It was observed that the specimen with the design for uniaxial tension could be deformed suitably using just one winding of the coil, while the wider specimens require multiple windings. The limit to the number of windings is explained by Psyk et al. [26] with the increase in inductance of the coil as the number of windings is increased. 

#### 2.1.3. Die Design

**Figure 8:**
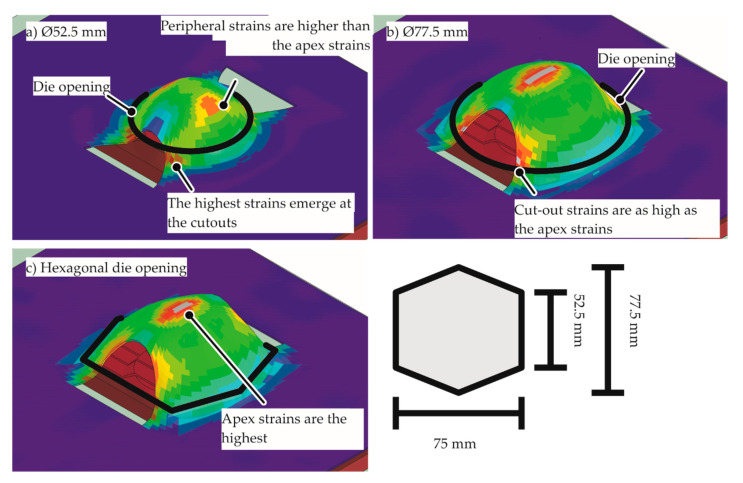
Simulation result with the plane strain specimen. The red contour indicates the highest equivalent plastic strain (**a**) with the standard Ø52.5 mm die opening, (**b**) with a Ø77.5 mm die opening, (**c**) with an optimized hexagonal die opening.

The standards of the geometry of the die are provided in ISO/DIS 12004–2 [25]. However, from the numerical modeling, it was observed that the highest strains were not present at the apex for the plane strain coil–specimen setup. The highest strains for this case were observed at the cutouts and the periphery of the specimen, as seen in Figure 8. To avoid these problems, the geometry of the die was changed. Firstly, by increasing the die opening diameter, the peripheral strains were removed, but the cutout strains were increased. Then, the cutout strains were decreased by changing the die opening shape to a hexagon and by optimization of its dimensions. 

### 2.2. Experimental Setup and Measurement Systems

**Figure 9:**
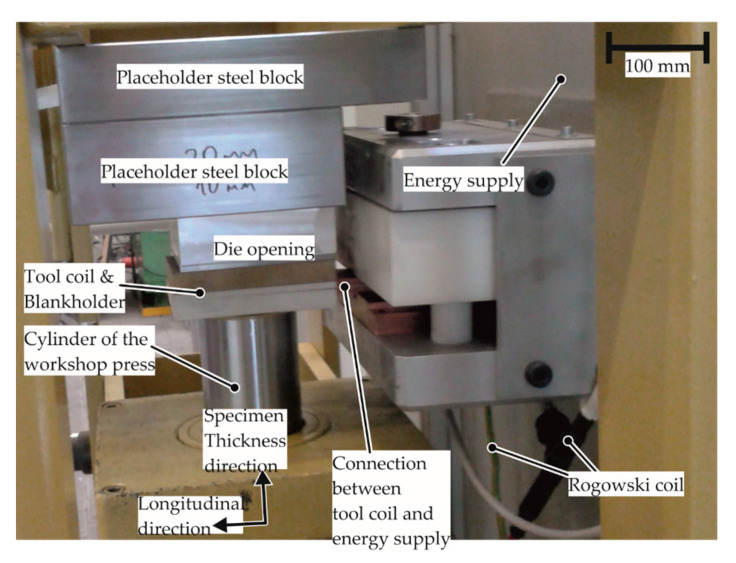
The setup of the electromagnetic forming limit test. The specimen is not seen; it is fixed between the tool coil and the die opening. A Photon Doppler Velocimeter (PDV) is also not seen; it is nestling in the uppermost steel block used as placeholder.

To determine the EM-FLCs, the experimental setup comprises of the specimen, a die opening, a tool coil, an energy supply, and a workshop press, as seen in Figure 9. The tool coil is connected to the energy supply, while the specimen, die opening, and the tool coil are held tightly by the press, which acts as the support and applies the blankholder force. The energy was supplied by a capacitor bank with the capacitance of 80 µF and an inner inductance of 40 nH. 

The current was measured using a Rogowski coil. For comparison of the experiments with the numerical model as well as estimation of the strain rate, the velocity of the apex of the specimen was measured using a Photon Doppler Velocimeter (PDV), the accuracy of which is discussed by Dolan [27]. A PDV sends a laser beam to the surface of the specimen and calculates the apex velocity based on the frequency of the reflected laser beams. The strain measurement is conducted optically using the system Aramis from the company GOM. The measurement is conducted using digital image correlation of the pictures taken before and after the forming process. The changes in the strain rate during the process could not be captured due to the very short duration of the process (less than 150 µs) and the low frame speed of the camera. For the fractured specimens, the fracture thickness was measured using scanning electron microscopy to determine the fracture limits.

### 2.3. Determination of Strain Rates for the Tests

As every geometry has a different strain rate at the apex for different discharge energies, the average strain rate for each test has to be estimated. This estimation of the strain rate was conducted using the velocity–time curves of the apex $v^{a}\left(t\right)$ measured during the experiments. Firstly, the velocity–time curve was converted to an apex height–time curve through the integration shown in Equation (2).

(2) $$h^{a}\left(t\right)=\;\overset{t}{\underset{0}{\int }}v^{a}\left(t\right)dt$$

**Figure 10:**
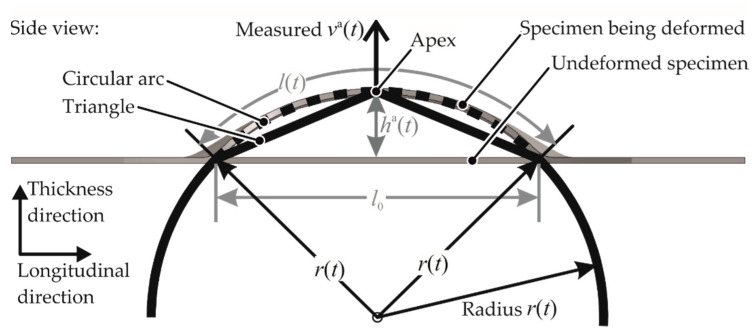
Apex velocity $v^{a}\left(t\right)$ was measured during each experiment. h(t) denotes apex height, l(t) denotes specimen length.

It was assumed that the specimen follows the shape of a circular arc of radius r(t) as shown in Figure 10. 

Then, the specimen length was expressed as a function of specimen height through the trigonometric relation in Equation (3).

(3) $$l\left(t\right)=\;\frac{\tan^{-1}\left(\frac{2h^{a}\left(t\right)}{l_{0}}\right)\left(4h^{a}{\left(t\right)}^{2}+l_{0}^{2}\right)}{2h^{a}\left(t\right)}$$

Then, the longitudinal engineering strain of the whole specimen (overall strain) is determined as:

(4) $${\overset{↔}{e}}_{\mathrm{long}}\left(t\right)=\;\frac{l\left(t\right)}{l_{0}}-1.$$

The circular arc assumption is not valid for the plane strain case, as the die geometry is hexagonal. The assumption was found to be invalid also in the case of biaxial strain, as the tool geometry in the longitudinal direction gives the specimen a triangular shape. In these cases, the basic procedure of defining the strain rate was the same, with the only difference that the longitudinal length was measured experimentally to get an empirical approximation of the function ${\overset{↔}{e}}_{long}\left(h(t\right))$.

For the conversion of the overall strain to apex strain $e_{\mathrm{long}}^{a}$, the strain distribution from the simulation is used. A strain concentration factor is defined, which varies for different materials and strain paths. This factor is further discussed in Section 4.2.1. This is shown in Equation (5).

(5) $$e_{\mathrm{long}}^{a}\left(t\right)=\;{\bar{k}}^{a}\;{\overset{↔}{e}}_{long}\left(t\right)$$

With the engineering strain $e_{\mathrm{long}}^{a}$ at the apex, all the logarithmic components and the von Mises equivalent plastic strain at the apex as a function of time can be calculated as shown in Equation (6).

(6) $$\varepsilon_{\mathrm{eqv}}^{a}\;\left(t\right)=\;\sqrt{\frac{2}{3}\left(\varepsilon_{\mathrm{long}}^{a}{\left(t\right)}^{2}+\;\varepsilon_{\mathrm{lat}}^{a}{\left(t\right)}^{2}+\;\varepsilon_{\mathrm{thick}}^{a}{\left(t\right)}^{2}\right)\;}$$

Then, the time course of the strain rate is calculated from Equation (7) as:

(7) $${\dot{\varepsilon^{a}}}_{\mathrm{eqv}}\left(t\right)=\;\frac{d\varepsilon_{\mathrm{eqv}}^{a}\;\left(t\right)}{dt}\;.$$

The average strain rate for each experiment was determined using Equation (8).

(8) $${\bar{\dot{\varepsilon }}}_{\mathrm{eqv}}=\;\frac{\int_{0}^{\varepsilon_{\mathrm{eqv},\mathrm{fin}}^{a}}{\dot{\varepsilon^{a}}}_{\mathrm{eqv}}\;d\varepsilon_{\mathrm{eqv}}^{a}}{\varepsilon_{\mathrm{eqv},\mathrm{fin}}^{a}}$$

where $\varepsilon_{\mathrm{eqv},\mathrm{fin}}^{a}$ represents the final von Mises equivalent plastic strain at the apex. As there is a very high variation of strain rate during the process, this provides an approximate average value for a particular FLC point. 

### 2.4. Validation and Usage of the Simulation Approach

**Figure 11:**
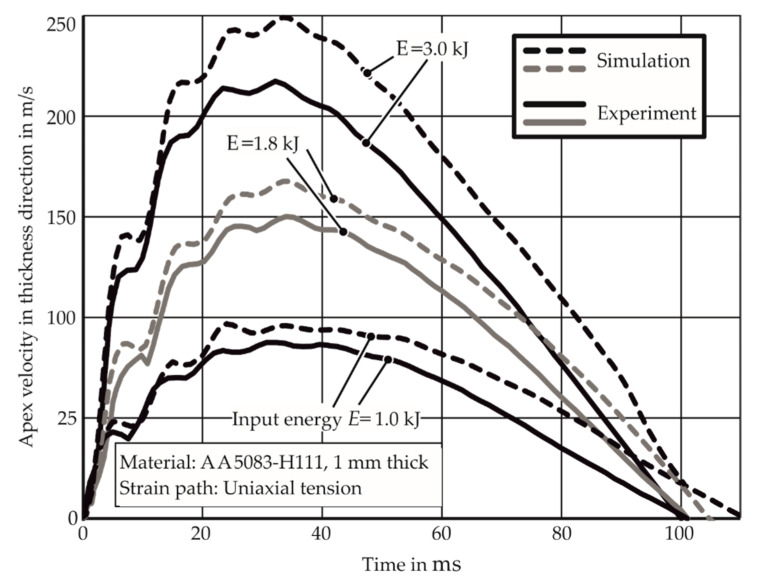
Measured and simulated specimen velocities during the electromagnetic forming limit test. E denotes input energy.

The apex velocity of the specimen is measured in the numerical model as well as the experiments using the PDV. It is observed in Figure 11 that the simulated velocities are higher than the experimental results as the numerical model does not consider strain-rate hardening. In addition, the difference between the experimental and the simulated velocities increases with the velocity increase in discharge energy. This is explained with the fact that the effect of strain rate hardening increases at higher strain rates.

**Figure 12:**
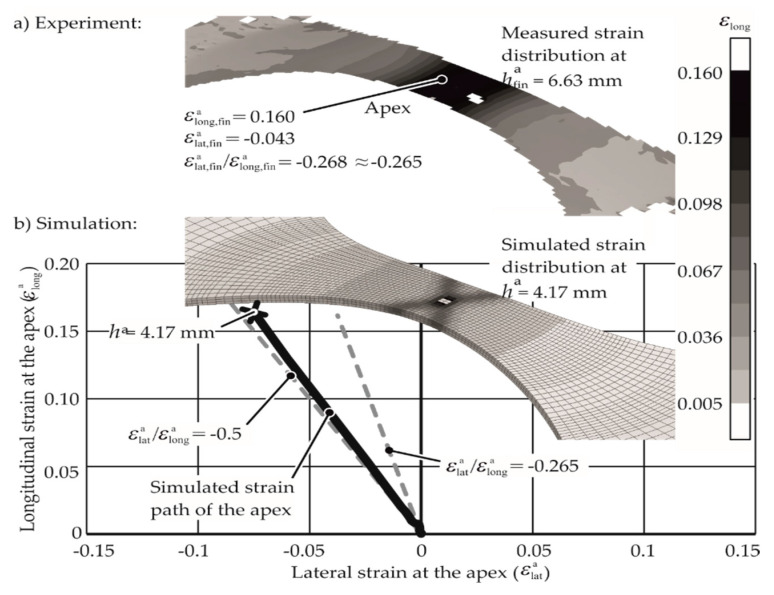
(**a**) Electromagnetic forming limit test with 1.0 kJ input energy, and (**b**) simulation of the same. Material: AA1050A-H24, 1 mm thick, specimen shape for uniaxial tension. $h^{a}$ denotes apex height.

A comparison of the strain paths for the material AA-1050a-H24 obtained from the numerical and experimental analyses is shown in Figure 12. The strain path in the numerical model is constant, but it is close to ε_lat,fin_/ε_long,fin_ of −0.5. The experimentally measured strain path is different from that of the numerical model due to the anisotropy in the material behavior, which is typical for most aluminum alloys. The results from the simulation are only used qualitatively, as it is observed that the simulation reflects the specimen deformation behavior appropriately. The history of the apex strains was examined in the numerical model to assure that the apex is deformed on a constant strain path.

## 3. Results

**Figure 13:**
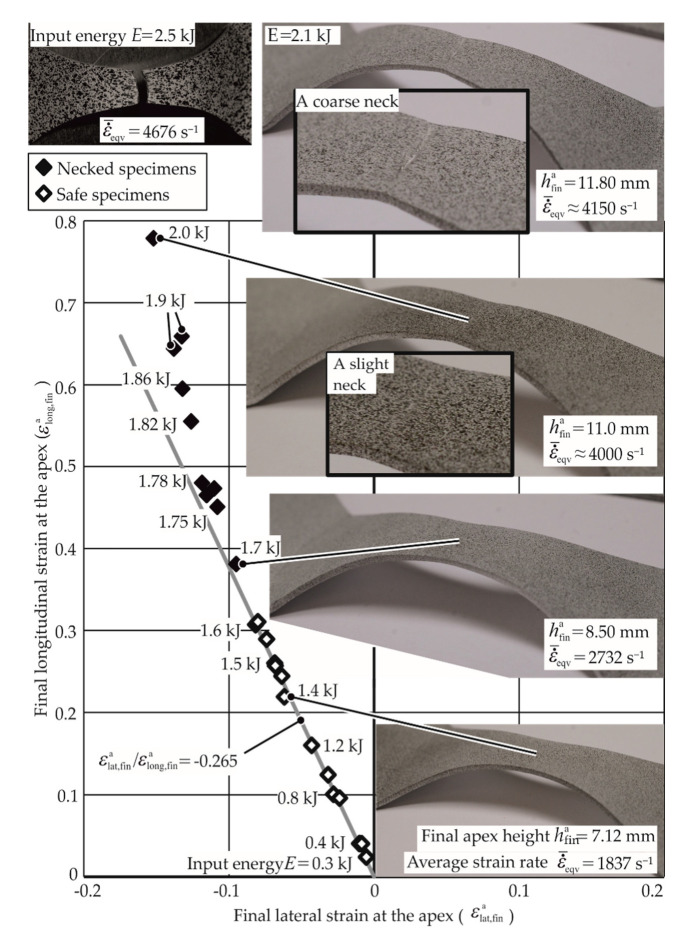
Electromagnetic forming limit test with the uniaxial specimen. Material: AA1050A-H24, 1 mm thick. Each diamond, empty or full, represents a separate experiment. The $\dot{\bar{\varepsilon_{eqv}}}$ is the average strain rate.

For particular specimen geometry, the discharge energy was increased until the experiment resulted in a necked or fractured specimen. For the uniaxial specimen of AA-1050a-H24, the strains along the strain path given by ε_lat,fin_/ε_long,fin_ = −0.265 are shown in Figure 13.

For the discharged energy until 1.7 kJ, no necking is seen. The specimens with discharge energies greater than 1.7 kJ fall away from the strain path. These specimens were observed to have necks, although the necks were extremely small and permitted strain measurement at the apex. With higher discharge energies (2.1 kJ for example), the necks became coarser, and strain measurement at the apex was not possible anymore. At 2.5 kJ, the specimen fractured at the apex. As the strains during the process are not known, the strains measured at the end of each experiment for various energies until necking are plotted to create the strain path. Furthermore, as seen from Figure 13, this strain path is constant until necking. The determination of apex strains for the specimens with apex fractures and no possibility for the measurement of the same is discussed in Section 3.1. 

### 3.1. Necking Limits for Tests Where Apex Strains Could Not Be Measured

**Figure 14:**
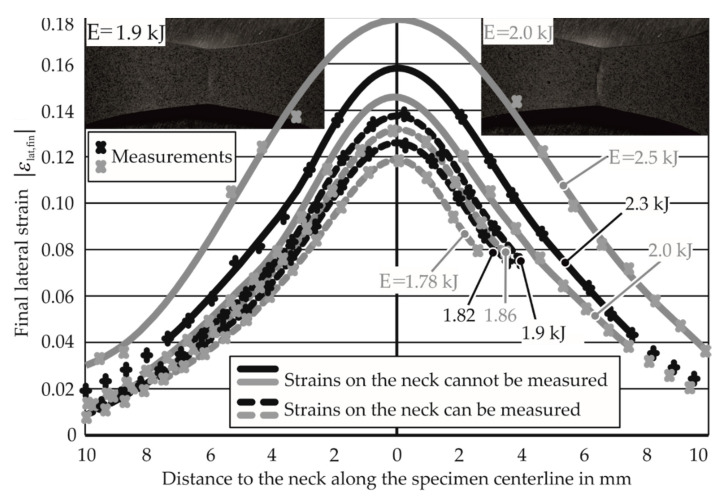
Curve fitting to the measured strains along the centerline. Each cross represents a measurement. E denotes input energy. Material: AA1050A, 1 mm thick, under uniaxial tension.

The measurement of the average strain rate for various energies and strain paths is discussed in Section 2.3. For the energies 1.78, 1.82, 1.86, and 1.9 kJ, the apex strains and the strains on the centerline were measurable and were plotted, as shown in Figure 14. For higher energies, the apex strains were not measurable and were estimated. A polynomial curve is fit through the lateral strains along the centerline for the lower energies and is used to determine the apex strains for the higher energy specimens, as explained in the standard quasi-static testing ISO/DIS 12004–2 [25]. For high-energy specimens, this provides the lateral strain at the stage where a neck has already developed at the apex. It is assumed that the lateral strain at the neck remains constant after necking. Based on this assumption, the lateral strain at the apex at the onset of necking is estimated. 

The strain at the apex in the thickness direction is found using the volume constancy assumption in plasticity. With the necking limits (ε_I,lim_, ε_II,lim_, ε_III,lim_) now known for all the specimens, the determination of strain rates for each discharge energy and strain path is required for the construction of the EM-FLCs. 

### 3.2. EM-FLCs for Different Average Strain Rates and Materials

**Figure 15:**
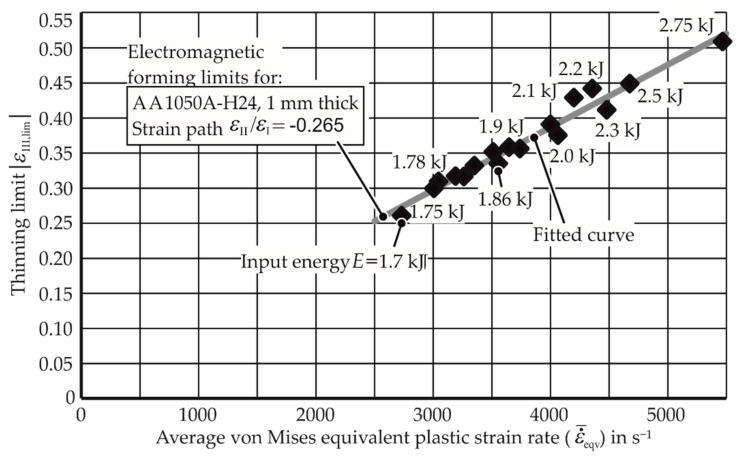
Electromagnetic forming limits of AA-1050a-H24 under uniaxial tension. Thinning limit is the amount of thinning at the onset of necking.

With the known average strain rates for the experiments and their necking limits in the thickness direction determined in Section 2.3 and Section 3.1, Figure 15 was drawn between the thinning limit and average strain rate for the strain path ε_II_/ε_I_ = −0.265. 

**Figure 16:**
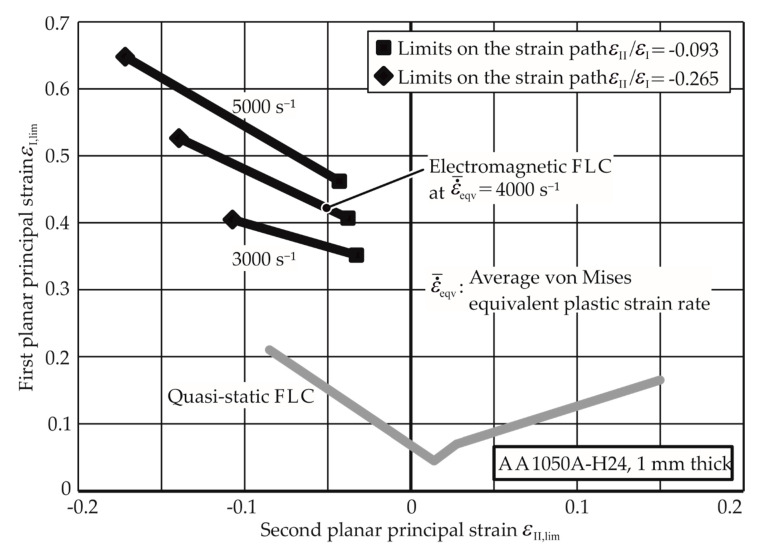
Electromagnetic forming limit curves (FLC) of AA-1050a-H2410 at different strain rates. The quasi-static FLC is given for comparison.

**Figure 17:**
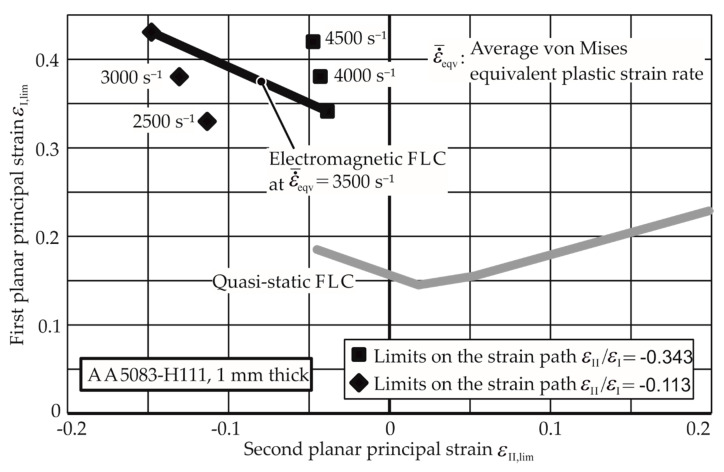
Electromagnetic forming limit curves (FLC) of AW-5083-H111 at different strain rates. The quasi-static FLC is given for comparison.

**Figure 18:**
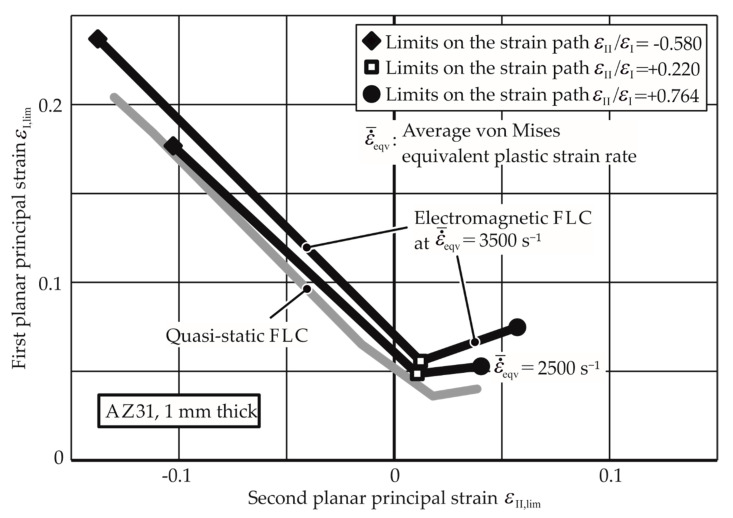
Electromagnetic forming limit curves (FLC) of Mg AZ31-O at different strain rates. The quasi-static FLC is given for comparison.

Through the linear fit curve for this path, the thinning limits for other average strain rates can be extrapolated. The higher EM-FLCs for strain rates 3000 s^−1^, 4000 s^−1^, and 5000 s^−1^ are plotted in Figure 16 with the quasi-static FLC for comparison. A detailed flowchart for the determination of the EM-FLCs can be founded in Appendix A. Figure 17 and Figure 18 show the higher strain rate EM-FLCs for the materials AW-5083-H111 and Mg AZ31-O. 

For the other strain paths (plain strain and biaxial tension) and other materials, the experiments were conducted as well. The results for AA-1050a-H24 are shown in Figure 16. It is observed that the biaxial specimens did not neck in the experiments, and hence, their necking limits could not be determined, as the energy required for the necking is relatively high and caused insulation problems in the experimental setup. However, it can be inferred that the necking limit for the biaxial tension strain path is very high at higher strain rates. 

For similar reasons, necking limits for specific strain paths for AW5083 were also not determined. For Mg AZ31-O, necking at all the strain states was possible, but the EM-FLCs are rather close to the quasi-static FLC, indicating that the formability of the magnesium alloy is not very strain rate-sensitive.

**Figure 19:**
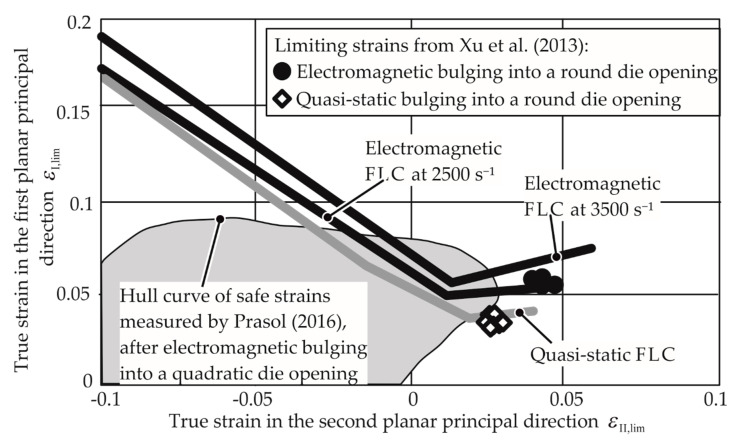
Results of Xu et al. [28] and Prasol [29] are compared with the EM-FLCs determined in this work. Mg AZ31-O, sheet thickness 1 mm.

It is observed that the EM-FLCs are much higher than the quasi-static FLCs for the aluminum alloys AA-1050a-H24 and AW-5083-H111. These results cannot be compared to the previous research, as their forming limits at specific average strain rates are not found in the literature, especially in free forming. However, the EM-FLCs for Mg AZ31-O can be compared to the previous research. As seen in Figure 19, the results from Xu et al. [28] agree with the current research. The experiments conducted by him were for the biaxial tension strain state. Due to the similarity of the process, it is assumed that the strain rates in their research must lie between 2500 and 3500 s^−1^. 

The strains reached by Prasol [29] partly exceed the limits obtained in the current research. The experiments were conducted by bulging the sheet electromagnetically into a quadratic die. The hull curve in Figure 19 shows the safe strains. However, the strain rates and the paths are not known for those experiments. It is essential that a constant strain path must be followed to determine a compatible EM-FLC, as the strain rate can provide different necking strains if a non-constant path is followed.

## 4. Discussion

### 4.1. Assumptions in EM-FLC Calculations

The lateral necking strain of a specimen is estimated based on the final lateral strain in Section 3.1. As an explanation, the assumption that no plastic deformation takes place outside the neck after necking is considered. This assumption dictates a constant specimen width outside the neck. This is also explained in the definitive book by Marciniak [20]: “Geometric constraint requires that the strain increment along the neck must be equal to that in the same direction just outside it … therefore the strain increment in the y-direction must be zero at all times.” The y-direction in the statement above is the lateral direction in the EM-FLC test. In case of an in situ measurement device where the apex strain–time course can be measured during the test, the apex strains at the start of necking could be directly measured, and no deduction from the final strain would be required.

The EM-FLCs are determined for an average strain rate of the process. This is essential, as the strain rate during the process inherently has a high variation. However, this may reduce the accuracy of the determined EM-FLCs, and they shall be therefore used with a reasonable safety factor. 

### 4.2. Reasons for Higher Forming Limits in EMF

#### 4.2.1. Strain Localization

It is known from quasi-static FLC determination that strain localization eventually leads to material failure. For this purpose, it is essential to examine the progress of strain concentration at the apex during quasi-static and EM-FLC determination tests. The overall strain concentration factor at the apex was defined as:

(9) $$k^{a}=\;\frac{e_{long}^{a}}{{\overset{↔}{e}}_{long}}.$$

**Figure 20:**
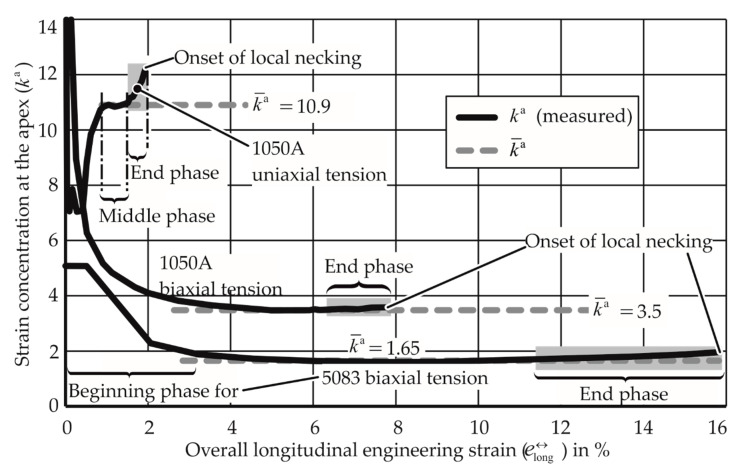
Strain concentration factor at the apex measured during quasi-static forming limit tests.

This quantity is used to relate the overall strain to the apex strain. This factor is determined experimentally for the quasi-static tests through in situ strain distribution measurement. For EM-FLCs, this was not possible, as the strain distribution was measured only after the process. In Figure 20, it is seen from the high $k^{a}$ value that the strain concentration at the apex at the beginning of the process is extremely high due to the underdeveloped contact between the punch and the die. In the middle phase of the experiment, $k^{a}$ is stable. This means that the factors that are working in favor of and against strain localization at the apex are in equilibrium. The factors that are working in favor of strain localization at the apex are the specimen thinning, the reduction in specimen width, and the applied displacement field. The factors that are working against strain localization are strain hardening, strain rate hardening, and inertial stabilization. In the middle phase of the experiment, the factors against localization are dominant, so a $k^{a}$ does not increase prominently. As the deformation progresses, the factors favoring the strain localization become increasingly dominant, leading to the increase of $k^{a}$ with an increasing rate as well. With further strain concentration, a local neck emerges as well. The constant value in the middle phase is assumed as the overall strain concentration factor of the experiment, which is specific to the material and the strain path.

**Figure 21:**
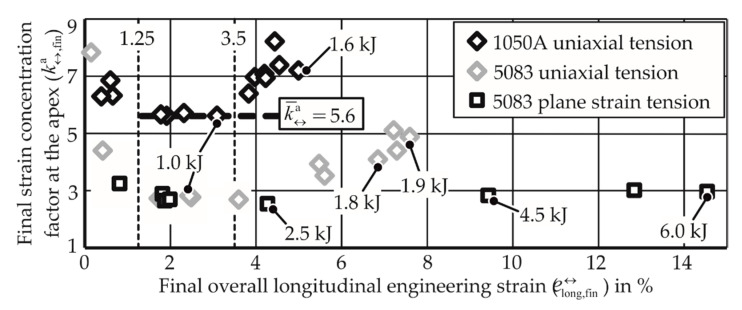
Strains measured after electromagnetic forming limit tests. Each point represents a test. Values in kJ reveal the input energy of the test.

The development of strain concentration in the electromagnetic process is hypothesized to be analogous to the quasi-static process. In the beginning phase, $k^{a}\;$ is very high due to the underdeveloped electromagnetic force field. The middle phase sees a relatively constant value, and very high values are observed toward the end, leading to local necking. From the experiments, Figure 21 is plotted for specific materials and strain paths.

While the strain paths in the quasi-static case belonged to the measured strains during the process, the points in the EM-FLC test all belong to different tests performed at different energies. 

From Figure 21, it is observed that for the uniaxial tension of AA-1050A, the specimens that reached strains between 1.25% and 3.5% have similar strain concentration factors, which is analogous to the middle phase of the quasi-static FLC test. 

**Figure 22:**
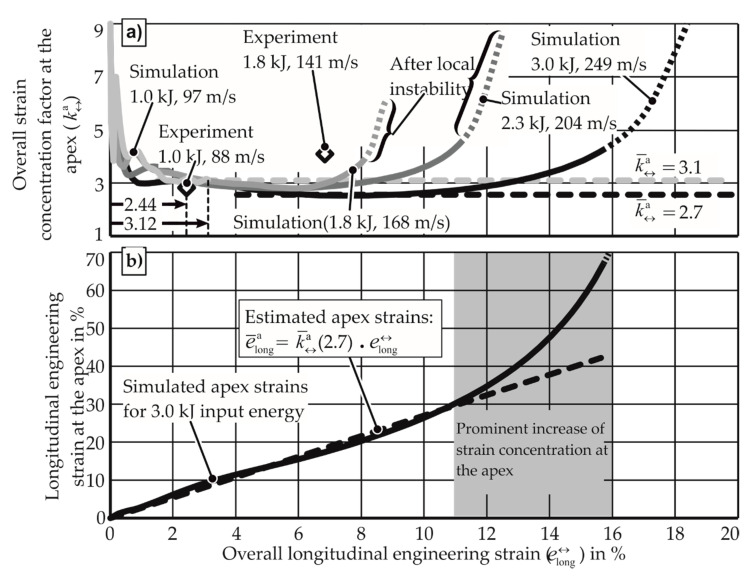
Electromagnetic forming limit tests with AW-5083 under uniaxial tension. (**a**) Strain concentration at the apex, (**b**) strain at the apex. kJ values give the input energy of the experiment, m/s values give the maximum apex velocity.

In Figure 21, the apex longitudinal strains for various discharge energies were measured after the experiments. While the strains themselves show compatibility with the three-phase scenario discussed, the hypothesis requires that strain concentration be determined from strains measured during the experiment. For this purpose, the simulations were performed for the given materials, as the strains at the apex of the specimen can be observed during the deformation. For AW-5083-H111, the simulations and the experiments for uniaxial tension provide the strain concentration factor, as seen in Figure 22. While the simulation overestimates the strain concentration factor (as strain-rate hardening is not considered), the qualitative behavior is similar to that of the quasi-static FLC test. If the constant value of the strain concentration factor is chosen with the assumption that the strain distribution remains constant until necking, the high values observed at the end of the process are underestimated. However, this is acceptable, as the strain concentration factor during proportional straining is used for calculations in Section 2.3. Here, the conversion of longitudinal engineering strain to apex strain is carried out before the necking. 

From Figure 22, it is also observed that the simulations with higher energy (and consequently higher strain rate) neck at a higher overall longitudinal strain compared to the smaller discharged energies. When comparing these simulations, it is observed that with the increase of strain rate:

1. $k^{a}$ reduces
2. The end phase of deformation (where $k^{a}$ rises again) starts at a higher overall strain

The smaller $k^{a}$ value for the higher strain rate simulations cannot be directly interpreted as resistance of high strain rate specimens to strain localization. The strain concentration factor $k^{a}$ is not only a material property but also depends on specimen geometry and the displacement field. In this case, the low value of $k^{a}$ can be explained by the high forces applied on the walls and base of the specimen in the high strain rate tests to reduce the localization at the apex. The difference in the electromagnetic force field at different strain rates also explains the delay in the end phase of deformation. To evaluate if the material’s resistance to strain localization has increased, one must examine the following parameters:

- Apex strain: to be observed as the strain concentration starts to increase. This indicates whether for higher strain rates, the strain concentration starts to increase at a higher value of apex strain or not.
- Increase of strain concentration per unit increase of apex strain: This indicates whether the strain localization happens at a slower rate for higher strain rates compared to low strain rate cases.
- Strain concentration at the onset of necking: This indicates if the material can tolerate a higher strain concentration before a local neck emerges.

**Figure 23:**
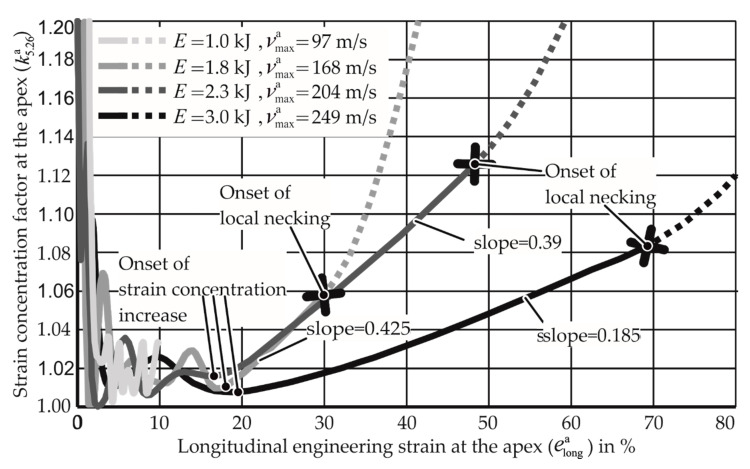
Simulations of the electromagnetic forming limit test with AW-5083-H111 under uniaxial tension. *E* denotes the input energy of the simulated experiment. $v_{max}^{a}$ denotes the maximum apex velocity in the simulation.

For the examination of the above parameters, four simulations with discharged energies varying from 1 to 3 kJ for AW-5083-H111 under uniaxial tension are performed, and graphs for the strain concentration factor at the apex are plotted against the longitudinal strain at the apex in Figure 23. The strain concentration factor examined here is denoted as $k_{5.26}^{a}$ as only 10 elements, having the length of 5.26 mm around the apex are examined for this analysis. It is observed from Figure 23 that after the initial unstable strain concentration until around 5% of the apex strain and the stable strain concentration until 20% of the apex strain, the increase of the strain concentration for all the specimens takes place at almost the same value of apex strain. 

It can also be seen that when the instability arises, the slope of the curve changes and becomes steeper, indicating local necking. The increase of strain concentration per unit increase in apex strain is observed using the slopes of the different curves. It is noted that for a high specimen velocity (strain rate), the slope is much lower than for the lower specimen velocity. The lower slope allows the apex to reach a higher apex strain without severe localization, thus increasing the formability. The development of the strain concentration at the apex indicated by this measure shows that it develops much slower for higher strain rates.

When the strain concentration at the onset of necking is compared, it is seen that for the higher strain rate specimen with the velocity of 204 m/s, the apex strain remains stable until the $k_{5.26}^{a}$ value of 1.12. This value is only 1.06 for the specimen velocity of 168 m/s. However, this increase in tolerance of $k_{5.26}^{a}$ values is finite, as the for specimen with the velocity 249 m/s, the material fails for a smaller strain concentration factor. 

#### 4.2.2. Fracture Surface Assessment

The assessment of the fracture surfaces of various specimens of AA-1050a-H24 from EM-FLC tests shows the presence of elongated dimples (structures resulting from void nucleation and growth) in the thickness and longitudinal direction. The presence of dimples in the fracture surface of the EM-FLC test specimens indicates that the failure mechanism is ductile at high strain rates.

**Figure 24:**
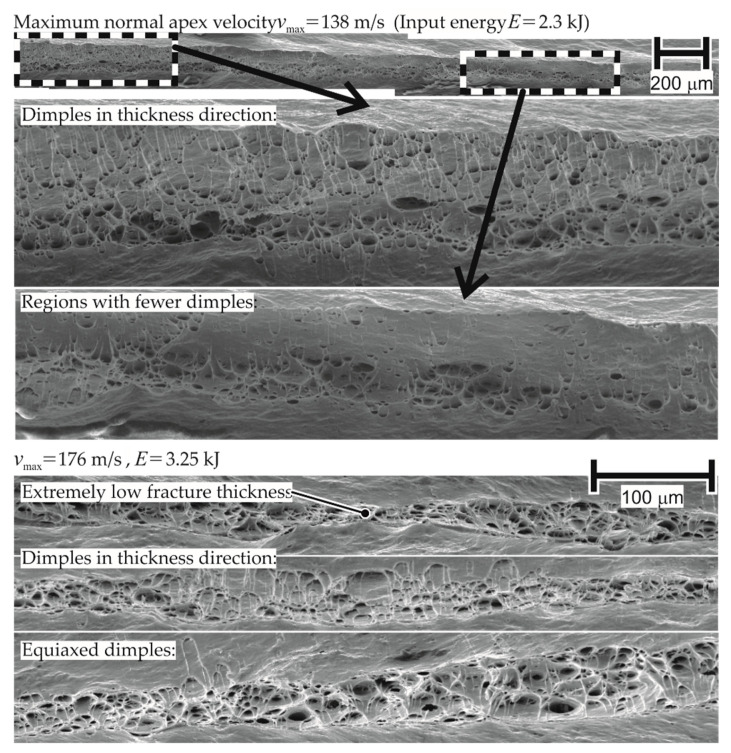
Fracture of electromagnetically formed 1 mm thick AA-1050a-H24 under uniaxial tension.

Such a fractured surface for a failed specimen in quasi-static testing showed the presence of equiaxed dimples only (dimples with similar dimensions in all directions). The presence of dimples elongated in the thickness direction indicates the existence of out-of-plane shear stresses, as these stresses are in the thickness direction and extend these dimples in the thickness direction. This is proposed as one of the reasons for higher forming limits in Section 4.2.3. The corresponding micrograph is shown in Figure 24. 

**Figure 25:**
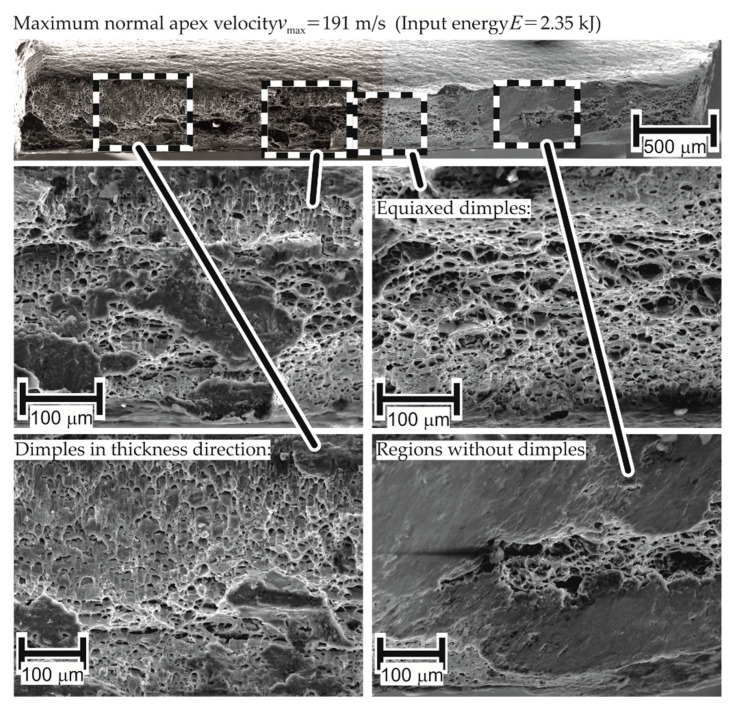
Fracture of electromagnetically formed AA5083-H111 under uniaxial tension (initial thickness 1 mm).

On observation of the fracture surface of AW-5083-H111 in Figure 25, certain regions without dimples are noticed. Based on the reasoning used for inferring that the failure for AA-1050a-H24 is ductile at high strain rates, the absence of dimples implies a change in the failure mechanism to brittle for AW-5083-H111. However, the current work considers an alternative explanation as well: such surfaces may result from the rubbing of mating fracture surfaces at high temperatures, which is also seen in the research of Shih-Chieh and Duffy [30]. While the fracture surface provides useful information about the forces during the process, it cannot be explicitly stated that the change in failure mechanism is the reason for the difference in material properties at higher strain rates when compared to quasi-static tests.

#### 4.2.3. Out-of-Plane Shear Stresses

**Figure 26:**
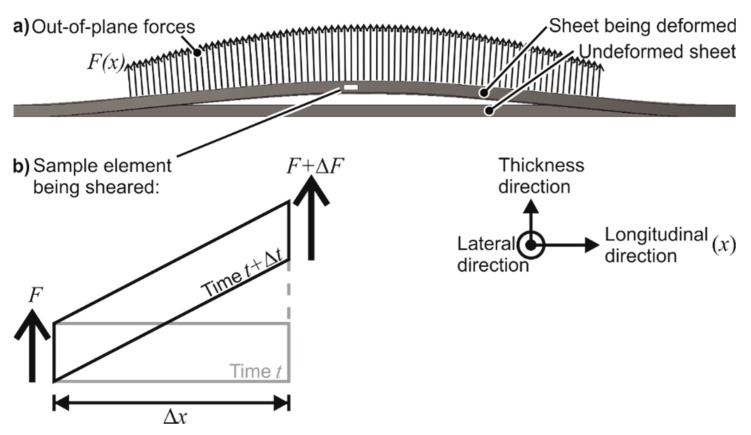
Out-of-plane volumetric forces can induce out-of-plane shear.

From the fracture surfaces of AW-5083-H111, it was observed that the quasi-statically formed dimples are elongated in the lateral direction and are hence driven by in-plane shear stresses. The elongation of the dimples in the longitudinal and thickness directions in EMF shows that in electromagnetic forming, the failure is driven by tensile and out-of-plane shear stresses. The out-of-plane shear stresses are absent in quasi-static forming. The reason for the presence of out-of-plane shear stresses is proposed as electromagnetic forces (Lorentz forces), as shown in Figure 26. These forces are out-of-plane in nature, primarily leading to in-plane stretching. If such forces are applied slowly (as in the quasi-static case), the element shown in Figure 26b will be rotated. Due to the high speed of the process, the inertia of the element becomes a participating factor, and shearing of the element is preferred. 

The claim that out-of-plane shear stresses lead to higher forming limits is attested by previous research about other high-speed processes done by Eyckens et al. [31]. Using a Marciniak–Kuczynski [20] analysis, the necking limits of an isotropic sheet were increased. Allwood and Shouler [32] confirmed this prediction experimentally by the application of out-of-shear forces during the tensile test of 1 mm thick specimen of AA-1050a-H24, which could by elongated by 300%, while the possible elongation without the application of out-of-plane shear forces was only 8%.

The theory was attempted to be verified using numerical analysis. The simulations with the discharge energies 1, 1.3, 2.3, and 3 kJ were examined for the presence of out-of-plane shear stresses at the apex. It is observed that in the fastest simulation, the magnitude of out-of-plane shear stress reaches 30 MPa, which is significant with respect to the initial yield stress of the material (150 MPa). The magnitude of the shear stress also increases with the increase in specimen velocity. The existence of out-of-plane shear stresses is confirmed by the numerical simulations and is therefore presented as one possible reason for the increase in formability in EMF. 

#### 4.2.4. Higher Limits of Mg AZ31-O

The failure mechanism in quasi-static and electromagnetic forming could not be clearly distinguished for Mg AZ31-O using fracture surface analysis. An additional analysis was performed for the comparison of the deformation mechanisms in the two cases.

**Figure 27:**
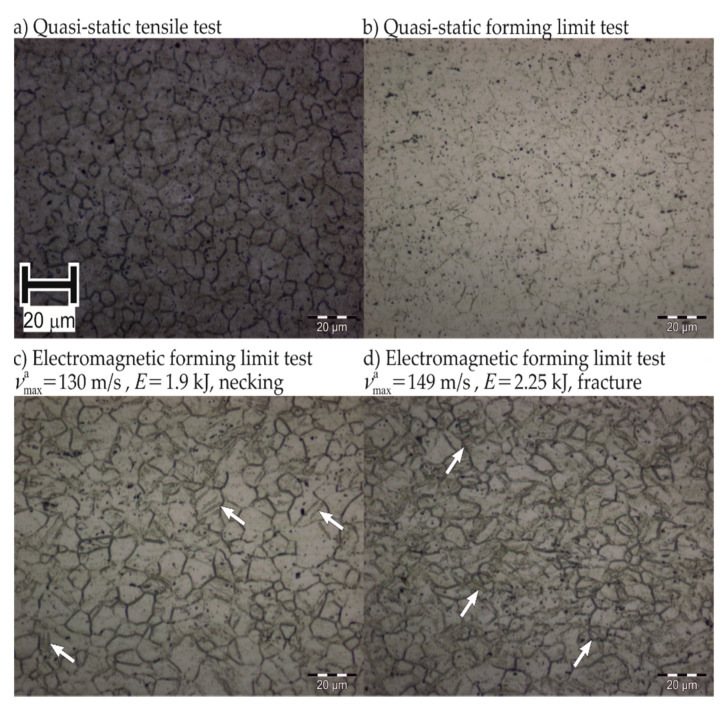
Polished micrographs of Mg AZ31-O deformed under uniaxial tension. Arrows show the twinning spots. *E* denotes input energy, $v_{max}^{a}$ denotes maximum apex velocity.

The polished micrograph of the fracture surface of the EMF specimen shows an abundance of twins in Figure 27, which is not the case for quasi-static forming. This suggests that the deformation mechanism includes more twinning in electromagnetic forming. Such a change of deformation mechanism at high rates was previously observed by Ferreira et al. [22], Ulacia et al. [23], and Li et al. [24].

However, these observations were made in a compressive load case. The mechanism change from slip to twinning contributes to the low formability enhancement in EMF of Mg AZ31-O compared to the formability increases observed for the aluminum alloys.

## 5. Conclusions

This work proposes a novel method to determine the forming limit curves for the electromagnetic forming process following the same requirements as the Nakajima test for quasi-static FLC determination. For the materials AA-1050a-H24, AW-5083-H111, and Mg AZ31-O, the method determines partially or fully the strain-rate dependent EM-FLCs. These FLCs showed that the electromagnetic free-forming necking limits are higher than the quasi-static ones. 

To explain the higher forming limits in EMF, besides the contributions of strain-rate hardening and inertia, the presence of out-of-plane shear stresses are proposed as a possible reason. The presence of out-of-plane electromagnetic forces applied at very high rates leads to significant magnitudes of the out-of-plane shear stresses. 

To determine the usefulness of the determined EM-FLCs, verification-based studies with real-life applications can be planned. As the primary advantage of EMF is the manufacturing of sheet parts in which the material is required to achieve higher strains than can be done in any quasi-static forming process, a geometry must be selected to be manufactured electromagnetically, and the EM-FLCs determined in this study must be used in process design as the failure criterion. Then, the numerical modeling with EM-FLCs as the limit must be verified experimentally. Furthermore, the determination of strain rate and strain distribution at the apex done in situ with the help of high-speed cameras will be beneficial for a greater understanding of the effect strain rates on EM-FLCs.

## Figures and Tables

**Figure A1:**
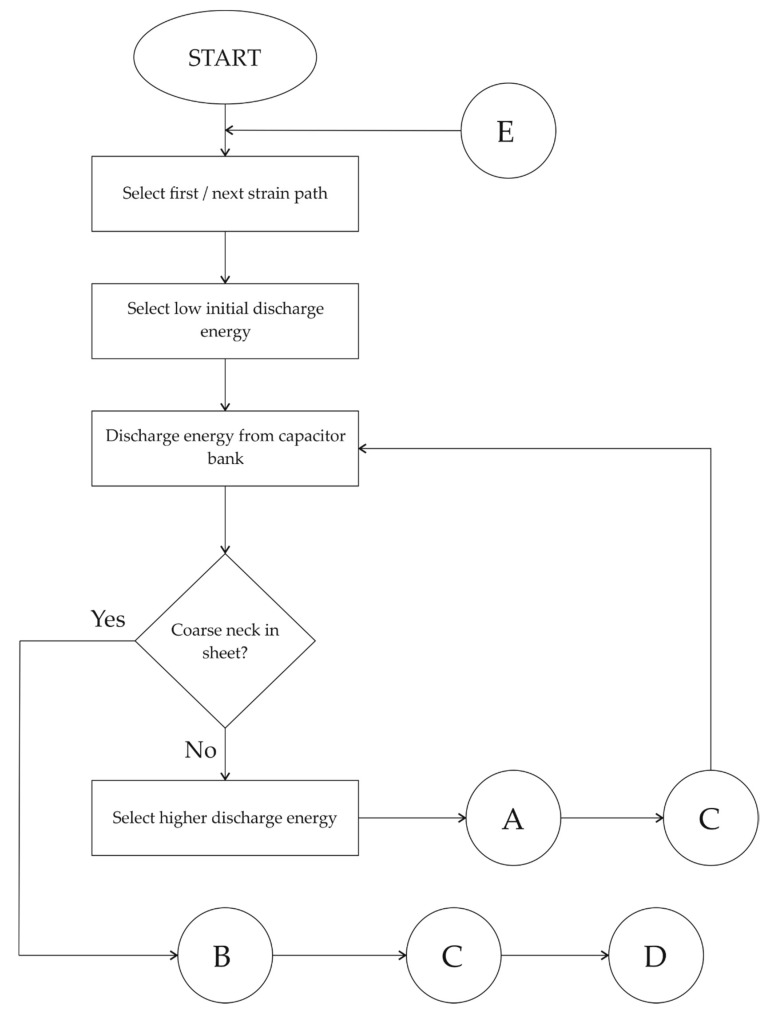
The procedure to determine the EM-FLCs. The flow continues via element D in Figure A4.

**Figure A2:**
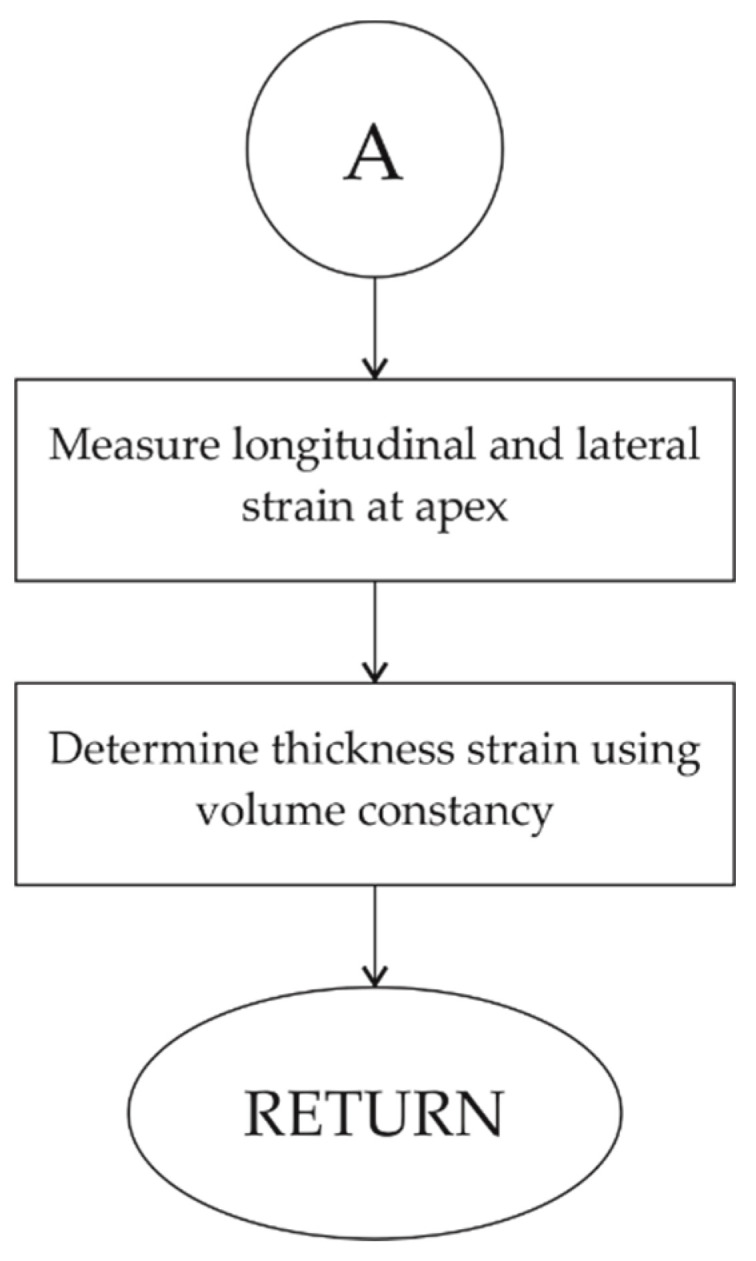
Apex strains for specimens without coarse necks.

**Figure A3:**
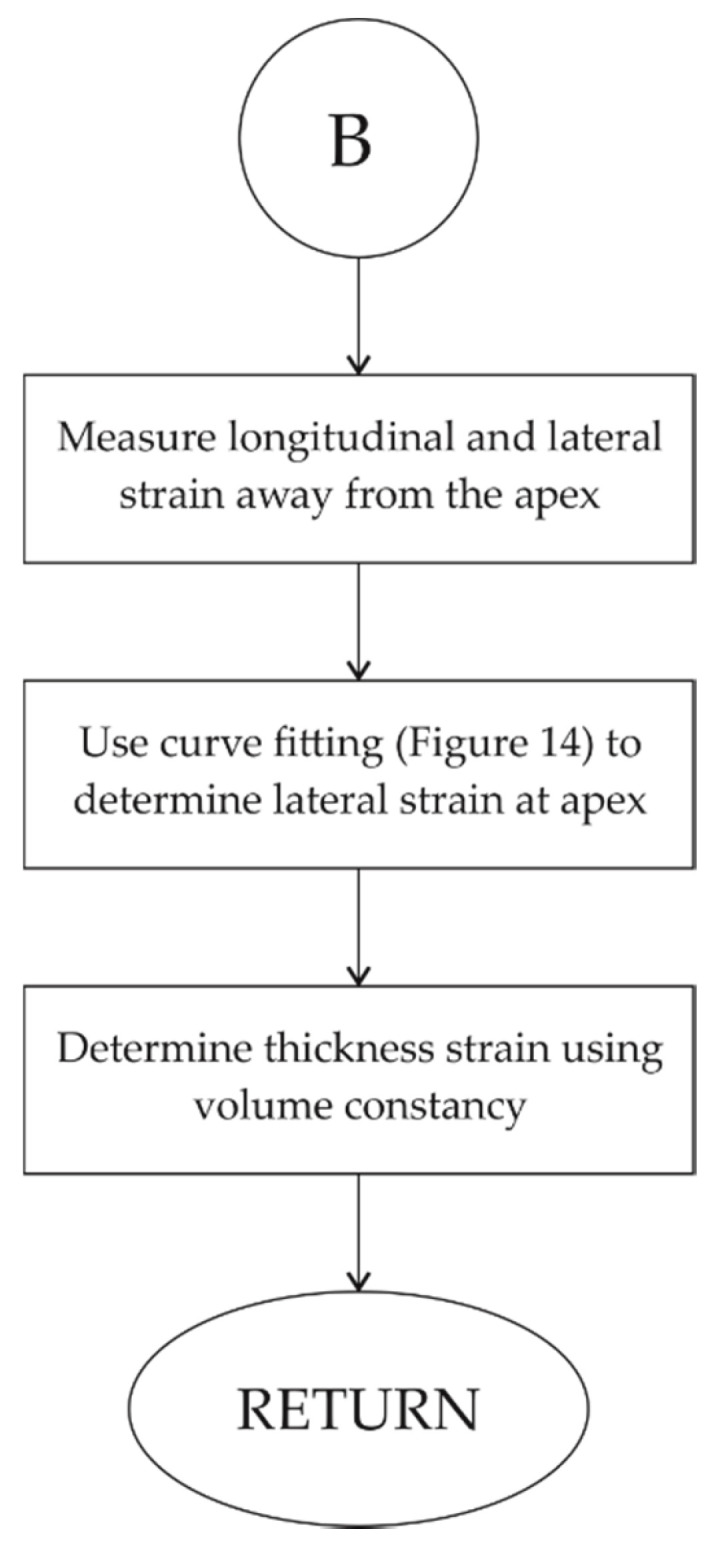
Apex strains for specimens with coarse necks.

**Figure A4:**
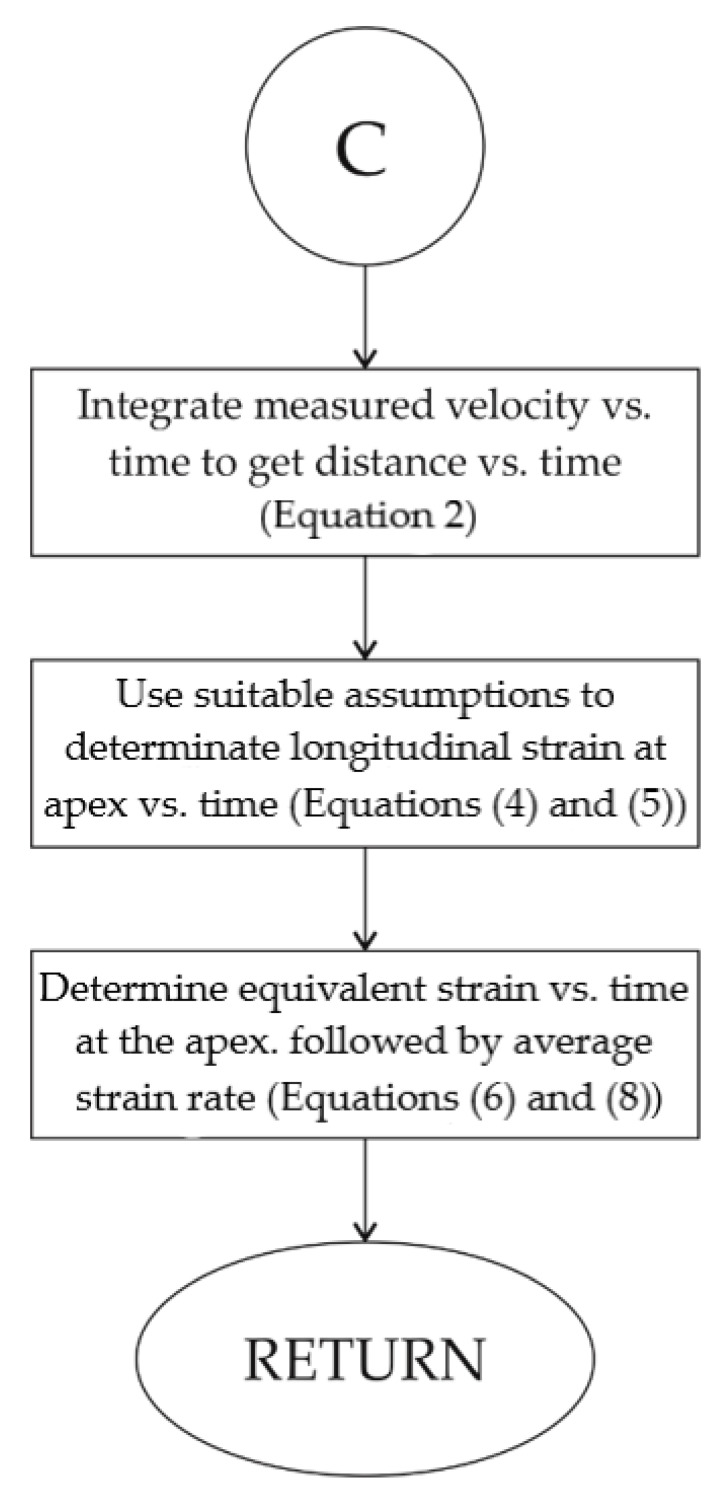
Continuation of the main routine.

**Figure A5:**
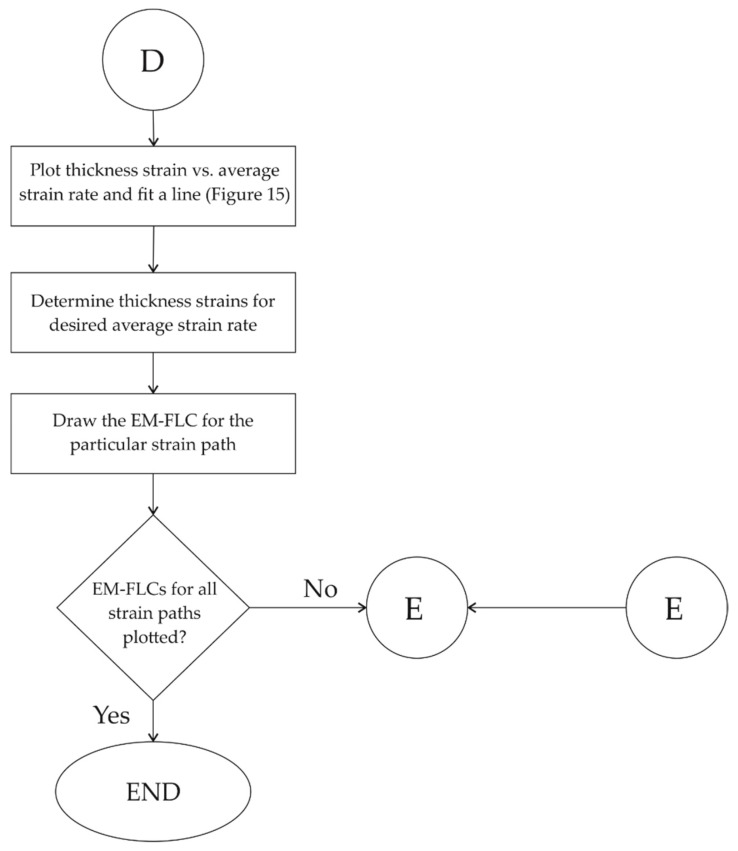
Average strain rate determination at the apex.

## Appendix A

Flow chart showing the detailed procedure to determine the EM-FLCs. The main routine begins in Figure A1 and continues in Figure A5. Figure A2, Figure A3 and Figure A4 are subroutines for the determination of apex strains and average strain rate at the apex, respectively.
